# Cultural adaptation of Internet- and mobile-based interventions for mental disorders: a systematic review protocol

**DOI:** 10.1186/s13643-020-01438-y

**Published:** 2020-09-03

**Authors:** Kerstin Spanhel, Sumeyye Balci, Harald Baumeister, Juergen Bengel, Lasse B. Sander

**Affiliations:** 1 Department of Rehabilitation Psychology and Psychotherapy, Institute of Psychology, University of Freiburg, Engelbergerstr. 41, 79085 Freiburg, Germany; 2 Department of Clinical Psychology and Psychotherapy, Institute of Psychology and Education, Ulm University, Lise-Meitner-Str. 16, 89081 Ulm, Germany

**Keywords:** Mental health gap, Cultural sensitive psychotherapy, eHealth, Low-threshold intervention

## Abstract

**Background:**

Internet- and mobile-based interventions (IMI) are an effective and scalable low-threshold solution to reach people who are undersupplied by current healthcare. Adapting interventions to the cultural and ethnic background of the target group enhances their acceptance and effectiveness. However, no systematic approach to cultural adaptation of IMI has been established so far. Therefore, this review aims to summarise components and procedures commonly used in the cultural adaptation of IMI for mental disorders, as well as the current evidence base on whether such a cultural adaptation leads to an increased acceptance, adherence, and effectiveness of IMI for mental disorders.

**Methods:**

A systematic literature search will be performed using the following databases: MEDLINE, Embase, PsycINFO, CENTRAL, and WoS. The search term will include keywords related to cultural adaptation, IMI, and mental disorders/disturbances. Two independent reviewers will evaluate studies against inclusion and exclusion criteria and extract study and intervention characteristics, details on the cultural adaptation approach, and outcome data. Quality of evidence will be assessed using the Quality Assessment Tool for Reviewing Studies with Diverse Designs, and results will be synthesised qualitatively.

**Discussion:**

Providing adequate mental healthcare regardless of cultural backgrounds is a major global health challenge. The planned systematic review will lay the foundation for the further development of the cultural adaptation of IMI for mental disorders by summarising the current state and providing recommendations for future research.

**Systematic review registration:**

PROSPERO CRD42019142320

## Background

Health inequalities remain a major global health challenge [1–3]. Particularly, migrants living in high-income countries and the general population of low- and middle-income countries (LMIC) are affected by a reduced availability of health services. This holds particularly true for mental health: although migrants often face various stressors before, during, and after migration [4], it has been found that they are less likely to use mental health services and, if they use them, find them less helpful [5, 6]. Similarly, a tremendous treatment gap in mental healthcare in LMIC has been revealed, i.e. the prevalence of mental disorders highly outnumbers the use of health services. It is estimated that, in LMIC, about 76–85% of people with a serious mental disorder do not receive any treatment, whereas about 35–50% receive no treatment in high-income countries [7–9]. Reasons for these mental health inequalities are linked to structural as well as individual barriers. Structural barriers include a small number of available mental health services and a poor accessibility of existing services [10–12]. Individual barriers include language and cultural barriers (e.g. understanding of disease and treatment processes, stigmatisation of mental disorders), a lack of information about the healthcare system, and a negative attitude towards the healthcare system [10–13].

The World Health Organization has called to take action in order to adequately reach these specific populations that migrated from or live in LMIC and to hereby achieve an improved global health equality [9]. This goal can only be reached when both individual and structural barriers are addressed. To reduce structural barriers, Internet- and mobile-based interventions (IMI) have been suggested as a low-threshold solution that enhances the accessibility of health services for a broad range of people in need [13–20]. The advantages of IMI include their anonymity, temporal and local independence, easy accessibility, and scalability [21–25]. The effectiveness of IMI has been proven in prevention, treatment, and aftercare for various mental disorders [25–29]. However, IMI are mostly developed and evaluated for people living in high-income countries, and they seem to be less effective for people with a differing ethnic background [30]. Thus, individual barriers to IMI have to be addressed simultaneously. Cultural adaptation of psychological interventions [31–34], i.e. the consideration of “language, culture, and context in such a way that it is compatible with the client’s cultural patterns, meanings, and values” ( [35]; p. 362), has been suggested to reduce existing individual barriers [36, 37] and to enhance their acceptance, relevance, and effectiveness [38–41]. Thus, culturally adapting IMI may be key to increase their reach and impact in culturally diverse people [19, 42]. In fact, in recent years, various research groups have culturally adapted IMI for people with diverse cultural backgrounds [43–47], which has been found to enhance the effectiveness of the IMI in the target groups [48]. Most research groups hereby relied on frameworks for the cultural adaptation of face-to-face treatments [31, 49–51], which, however, may not be valid when adapting IMI [52]. Previous reviews and meta-analyses have summarised studies that conducted IMI in LMIC [53] and investigated the extent and effectiveness of cultural adaptation of IMI for the treatment of common mental disorders among people with diverse cultural backgrounds [48]. Yet, the procedure of the cultural adaptation is often only poorly reported [43, 52, 54–56]. This makes it difficult to identify components of the cultural adaptation that may contribute to an enhanced acceptance and effectiveness of IMI, such as the language, illustrations, or example characters [43, 54, 56, 57].

Hence, in this review, we will systematically identify and summarise methods previously used for the cultural adaptation of IMI for mental disorders. We will answer the following research questions:

1. Which components are commonly considered in the cultural adaptation of IMI for mental disorders?
2. Which are procedures that are commonly used in the cultural adaptation of IMI for mental disorders?

Furthermore, we will summarise the current evidence base on whether cultural adaptation of IMI for mental disorders leads to an increased acceptance, adherence, and effectiveness of such interventions.

Thus, this review will inform future researchers and developers working on the cultural adaptation of IMI for a mental disorder. The cultural adaptation of IMI holds great potential to reduce both individual and structural barriers to treatment-seeking, which might contribute to reduce global mental health inequalities.

## Methods/design

This protocol has been developed in line with the Preferred Reporting Items for Systematic Review and Meta-Analysis Protocol (PRISMA-P) statement [58] (see Additional file 1). The systematic review has been registered with the International Prospective Register of Systematic Reviews (PROSPERO) database (https://www.crd.york.ac.uk/PROSPERO; registration number: CRD42019142320), and the findings will be reported using the Preferred Reporting Items for Systematic Review and Meta-Analysis (PRISMA) guidelines [59].

**Table 1:** Study inclusion criteria

Participants	(a) People with a cultural (i.e. national or ethnic) background differing from the initial target group of the intervention
Intervention	(b) Adapted to the target group (c) Psychological methods to address mental disorders/disturbances (d) In an Internet-, computer-, or mobile-based setting
Comparator	(e) With/without a control group
Outcomes	(f) Illustration of the cultural adaptation approach
Study type	(g) All study types (e.g. with/without follow-up, qualitative/quantitative)
Setting	(h) No restrictions in the type of setting
Report characteristics	(i) No restriction in the publication year (j) No restriction in language (k) Peer-reviewed journal articles

### Eligibility criteria

Studies will be chosen in line with the criteria outlined below and in Table 1.

Studies are eligible for inclusion (a) if the target group differs from the target group of the original intervention in terms of culture, i.e. nationality or ethnicity. (b) Interventions must have been adapted to the target group. If the intervention was only translated but not otherwise culturally adapted, it will be excluded. (c) Interventions must address mental health issues and must be based on psychological interventions. The term psychological intervention is defined according to Kampling et al. ( [60], p.2): “Psychological interventions aim to recognise, improve or prevent distress by direct or interactive communication.” They include cognitive behaviour therapy, psychodynamic psychotherapy, behaviour therapy or behaviour modification, systemic therapy, third-wave cognitive behavioural therapies, humanistic therapies, integrative therapies, and other psychological-oriented interventions. General health promotion interventions will not be included. (d) Interventions must predominantly be provided in an Internet-, computer-, or mobile-based setting. (e) Studies both with and without a control group will be included in the review. (f) Studies will be included if an illustration of the methods to culturally adapt the intervention is provided in the manuscript or—upon request—by the authors. (g) All study types, i.e. controlled and non-controlled trials, qualitative and quantitative studies, studies with or without a follow-up assessment, will be included. (h) There will be no restrictions by type of setting. (i) Neither will there be a restriction in the publication year, (j) nor in the language. Articles will be translated to English. (k) Only peer-reviewed journal articles will be included to guarantee a high quality of the extracted data. We assume that the information in grey literature will not significantly affect our research questions, as sufficient information will be available in peer-reviewed literature. In order to save resources, grey literature will thus be excluded.

### Search strategy

Electronic databases will be searched to identify published work, including MEDLINE, PsycINFO, Cochrane Central Register of Controlled Trials (CENTRAL), Embase, and Web of Science (WoS). A comprehensive search strategy developed by the project team will be used, which will include medical subject headings (MeSH terms) and text words related to the key elements of cultural adaptation of interventions in an Internet-, computer-, or mobile-based setting. The search term will include keywords of a parallel systematic review for the cultural adaptation of IMI in the context of health promotion. The search will not be limited. We used a validation set of 13 studies to test the sensitivity of the search term. The test search yielded a coverage of 100%. The full search term for PsycINFO can be found in Additional file 2. To complement the search, backward searches of the reference lists as well as forward searches of the identified relevant publications will be conducted.

### Study records

#### Data management

Literature search results will be uploaded to covidence, a software for reference management that also facilitates the communication among the reviewers. It will be used to identify and remove duplicates, to categorise publications according to their inclusion or exclusion, as well as for data extraction and quality assessment.

#### Study selection process

The selection of articles will be conducted by two independent reviewers (KS, SB). In a first step, all titles and abstracts yielded by the search will be screened in duplicate against the inclusion criteria. Full texts of the articles of all titles and abstracts that seem to meet the inclusion criteria or of which there is any uncertainty will be obtained. In a second step, these full texts will be screened in duplicate in terms of the eligibility criteria. Disagreement on including articles will be resolved in discussion; if further needed, a third reviewer (LS) will be consulted. Thus, selected articles will be searched backward and forward; new articles will be screened in the alike way. Reasons for exclusion will be recorded. Neither of the reviewers will be blind to the journal titles or to the study authors or institutions.

**Fig. 1:**
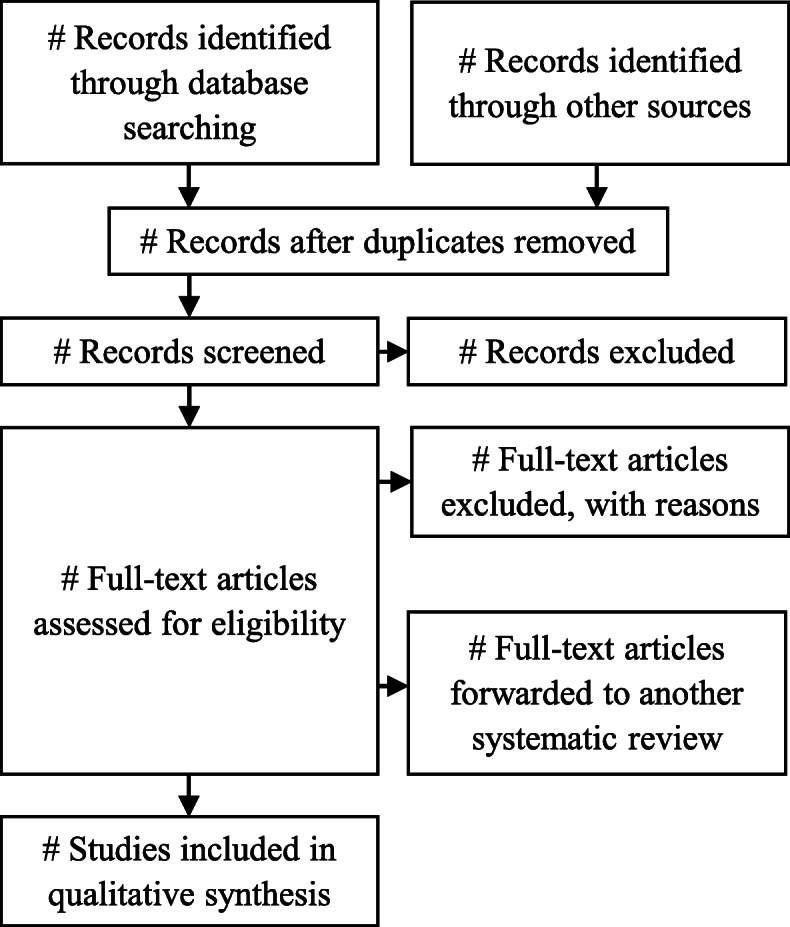
Study selection process (adapted from [59])

A PRISMA flow chart [59] will illustrate the study selection process (see Fig. 1).

#### Data extraction process

The following information will be extracted from each article by an independent reviewer (KS) using an extraction form, if the information is available: (a) study identification items (first author, year of publication, type of document), (b) sample characteristics (sample/group size, age, gender, country of origin, ethnicity), (c) study design characteristics (control group: no/non-adapted/delayed intervention or treatment as usual, randomisation, type of assessments, follow-up assessments), (d) study settings (country of study conduction, recruitment strategy), (e) original intervention characteristics (language, type, target group: gender/age/cultural background, target mental disorder, delivery mode, level of human support/guidance, duration of intervention and modules, components of intervention, signposting to other interventions), (f) culturally adapted intervention characteristics (language, type, target group: gender/age/cultural background, target mental disorder, delivery mode, level of human support/guidance, duration of intervention and modules, components of intervention, signposting to other interventions), (g) details on cultural adaptation approach (language, persons, metaphors, content, concepts, goals, methods, context, design, structure, functionality, aesthetics, open aspects), details on the process of cultural adaptation (feasibility/pilot trial, literature review, qualitative methods, persons involved), and illustration of an orientation towards a guideline of cultural adaptation of face-to-face treatments (name of authors, name of guidelines), as well as (h) outcomes (participation and attrition rates: maximum/minimum/median number of sessions completed, primary outcomes/secondary outcomes: which outcomes were used, tools used to measure outcomes, baseline symptom severity for all outcomes, findings/outcomes as measured by authors). We will contact the study authors to resolve any uncertainty and receive missing information, if the variables of interest are not available or not clearly presented in the studies. All extracted data will be double-checked by a second independent reviewer (SB).

### Quality assessment

Two independent reviewers (KS, SB) will assess the Quality Assessment Tool for Reviewing Studies with Diverse Designs (QATSDD) [61]. The quality of the following criteria will be evaluated: (1) explicit theoretical framework, (2) statements of aims/objectives in main body of report, (3) clear description of research setting, (4) evidence of sample size considered in terms of analysis, (5) representative sample of target group of a reasonable size, (6) description of procedure for data collection, (7) rationale for choice of data collection tool(s), (8) detailed recruitment data, (9) statistical assessment of reliability and validity of measurement tool(s) (quantitative only), (10) fit between stated research question and method of data collection (quantitative), (11) fit between stated research question and format and content of data collection tool (qualitative), (12) fit between research question and method of analysis, (13) good justification for analytical method selected, (14) assessment of reliability of analytical process (qualitative), (15) evidence of user involvement in design, and (16) strengths and limitations critically discussed.

The quality of the studies will be rated against the criteria on a four-point scale, producing a global score showing a low or high quality. If discrepancies arise between the two reviewers, they will be resolved in discussion; if further needed, a third reviewer (LS) will be consulted.

### Data synthesis

A narrative synthesis of all included articles will be conducted. Thereby, the results will be described in detail in text and tables, including the characteristics and findings of the studies (i.e. extracted data). Similarities and links between the included studies will be explored. The narrative synthesis will inform the first and second research questions by providing a qualitative report on the procedure of cultural adaptation and hereby considered components. Findings related to the effectiveness of, acceptance of, and adherence to the culturally adapted IMI for mental disorders will be displayed, if possible, in comparison to the non-adapted IMI.

### Meta-biases

If study protocols are available, we will compare the outcomes reported in the protocol and in the published report in order to examine the potential for selective outcome reporting. If not available, outcomes reported in the methods and result sections of the published reports will be compared.

## Discussion

Previous research suggests that culturally adapting psychological interventions [40, 41] as well as IMI [48] may enhance their effectiveness in populations differing from the initial target group. Whereas there are a number of guidelines for the cultural adaptation of face-to-face treatments, no consistent procedure exists in culturally adapting IMI. This systematic review will provide a summary of the cultural adaptation of IMI for mental disorders, which can enable an improved methodological procedure. Specific components and procedures that are suggested to be important to adapt will be identified by comparing previously conducted studies. Additionally, the acceptance and effectiveness of culturally adapted IMI for mental disorders will be investigated, and, if possible, compared to the non-adapted IMI. However, the heterogeneity in clinical, methodological, and statistical approaches may limit the interpretability. This review will include trials that may differ on the addressed mental disorders, cultural backgrounds of the target population, methods, interventions, control groups, assessments, and outcomes. Due to being a rather young research field and the linked assumption of a limited amount of relevant studies, we aim to conduct a broad review including heterogeneous studies. By contrast, studies in which culturally sensitive IMI for mental disorders were developed without the adaptation of an earlier version will not be included in the review; this is due to the assumption that the development of such an intervention requires other aspects than its adaptation.

The planned synthesis of the current state of cultural adaptation of IMI for mental disorders will lay the foundation for its further development, informing both researcher and developer of IMI. This will help in making IMI impactful for people living in or migrating from LMIC and contribute to delivering behavioural health services worldwide.

## Supplementary information

**Additional file 1.** PRISMA-P 2015 checklist.

**Additional file 2.** PsycINFO search term.

## Data Availability

Not applicable.

## Abbreviations

CENTRAL: Cochrane Central Register of Controlled Trials
IMI: Internet- and mobile-based interventions
LMIC: Low- and middle-income countries
MeSH: Medical subject headings
PRISMA: Preferred Reporting Items for Systematic Review and Meta-Analysis
PRISMA-P: Preferred Reporting Items for Systematic Review and Meta-Analysis Protocol
PROSPERO: Prospective Register of Systematic Reviews
QATSDD: Quality Assessment Tool for Reviewing Studies with Diverse Designs
WoS: Web of Science
