# Supplementary material for: Cultural adaptation of Internet- and mobile-based interventions for mental disorders: a systematic review protocol
Source: Syst Rev. 2020 Sep 3;9:207. doi: 10.1186/s13643-020-01438-y (PMC7472576; doi:10.1186/s13643-020-01438-y)
Supplement: Supplementary file 2 — Additional file 2. PsycINFO search term. [file 13643_2020_1438_MOESM2_ESM.docx]

**Additional file 2: PsycINFO search term**

| S1 | DE „internet“ |
| --- | --- |
| S2 | DE „Mobile Devices“ |
| S3 | DE „Cellular Phones“ |
| S4 | DE „Computers“ |
| S5 | TI computer |
| S6 | TI website |
| S7 | TI websites |
| S8 | TI web |
| S9 | TI online |
| S10 | TI internet |
| S11 | TI webbased |
| S12 | TI internetbased |
| S13 | TI media-based |
| S14 | TI computerized |
| S15 | TI computerised |
| S16 | TI cyber |
| S17 | TI mobile |
| S18 | TI App |
| S19 | TI Apps |
| S20 | TI smartphone |
| S21 | TI smartphones |
| S22 | TI phone |
| S23 | TI phones |
| S24 | S1 OR S2 OR S3 OR S4 OR S5 OR S6 OR S7 OR S8 OR S9 OR S10 OR S11 OR S12 OR S13 OR S14 OR S15 OR S16 OR S17 OR S18 OR S19 OR S20 OR S21 OR S22 OR S23 |
| S25 | DE „Psychotherapy” |
| S26 | DE „Analytical Psychotherapy” |
| S27 | DE „Behavior Therapy“ |
| S28 | DE „Brief Psychotherapy“ |
| S29 | DE „Cognitive Behavior Therapy“ |
| S30 | DE „Narrative Therapy“ |
| S31 | DE „Psychodynamic Psychotherapy“ |
| S32 | DE „Psychodynamic Psychotherapy“ |
| S33 | DE „Psychotherpeutic Counseling“ |
| S34 | DE „Behavior Modification“ |
| S35 | DE „Self-Management” |
| S36 | DE „Self-Instructional Training“ |
| S37 | DE „Self-Help Techniques“ |
| S38 | DE „Psychotherapeutic Techniques“ |
| S39 | DE „Cognitive Techniques“ |
| S40 | DE „Cognitive Restructuring“ |
| S41 | DE „Cognitive Therapy“ |
| S42 | DE „Therapeutic Processes“ |
| S43 | DE „intervention“ |
| S44 | TI Intervention OR AB Intervention |
| S45 | TI Interventions OR AB Interventions |
| S46 | TI treatment OR AB treatment |
| S47 | TI treatments OR AB treatments |
| S48 | TI therapy OR AB therapy |
| S49 | TI therapies OR AB therapies |
| S50 | TI therapeutics OR AB therapeutics |
| S51 | TI therapeutic OR AB therapeutic |
| S52 | TI psychotherapy OR AB psychotherapy |
| S53 | TI psychotherapies OR AB psychotherapies |
| S54 | TI psychoeducation OR AB psychoeducation |
| S55 | TI psychotherapeutic OR AB psychotherapeutic |
| S56 | TI psychotherapeutics OR AB psychotherapeutics |
| S57 | TI cbt OR AB cbt |
| S58 | TI program OR AB program |
| S59 | TI programs OR AB programs |
| S60 | TI programme OR AB programme |
| S61 | TI programmes OR AB programmes |
| S62 | TI training OR AB training |
| S63 | TI trainings OR AB trainings |
| S64 | TI lesson OR AB lesson |
| S65 | TI lessons OR AB lessons |
| S66 | TI counselling OR AB counselling |
| S67 | TI counseling OR AB counseling |
| S68 | TI coaching OR AB coaching |
| S69 | TI self-help OR AB self-help |
| S70 | TI self-care OR AB self-care |
| S71 | TI selfcare OR AB selfcare |
| S72 | TI self-change OR AB self-change |
| S73 | TI self-instruction OR AB self-instruction |
| S74 | DE „Computer Assisted Therapy” |
| S75 | DE „Online Therapy” |
| S76 | DE „Telemedicine” |
| S77 | TI telemedicine OR AB telemedicine |
| S78 | TI telehealth OR AB telehealth |
| S79 | TI tele-health OR AB tele-health |
| S80 | TI telecare OR AB telecare |
| S81 | TI eHealth OR AB eHealth |
| S82 | TI e-health OR AB e-health |
| S83 | TI „electronic health“ OR AB „electronic health“ |
| S84 | TI „e-mental health“ OR AB „e-mental health“ |
| S85 | TI e-therapy OR AB e-therapy |
| S86 | TI e-therapeutic OR AB e-therapeutic |
| S87 | TI mHealth OR AB mHealth |
| S88 | TI m-health OR AB m-health |
| S89 | TI „mobile health” OR AB „mobile health“ |
| S90 | TI cCBT OR AB cCBT |
| S91 | TI iCBT OR AB iCBT |
| S92 | S74 OR S75 OR S76 OR S77 OR S78 OR S79 OR S80 OR S81 OR S82 OR S83 OR S84 OR S85 OR S86 OR S87 OR S88 OR S89 OR S90 OR S91 |
| S93 | S25 OR S26 OR S27 OR S28 OR S29 OR S30 OR S31 OR S32 OR S33 OR S34 OR S35 OR S36 OR S37 OR S38 OR S39 OR S40 OR S41 OR S42 OR S43 OR S44 OR S45 OR S46 OR S47 OR S48 OR S49 OR S50 OR S51 OR S52 OR S53 OR S54 OR S55 OR S56 OR S57 OR S58 OR S59 OR S60 OR S61 OR S62 OR S63 OR S64 OR S65 OR S66 OR S67 OR S68 OR S69 OR S70 OR S71 OR S72 OR S73 |
| S94 | S24 AND S93 |
| S95 | S92 OR S94 |
| S96 | DE "Cross Cultural Treatment" |
| S97 | DE "Cross Cultural Counseling" |
| S98 | DE "Multicultural Counseling" |
| S99 | DE "Cross Cultural Psychology" |
| S100 | DE "International Psychology" |
| S101 | DE "Transcultural Psychiatry" |
| S102 | DE "Cross Cultural Differences" |
| S103 | DE "Racial and Ethnic Differences" |
| S104 | DE "Cultural Sensitivity" |
| S105 | DE "Multiculturalism" |
| S106 | DE "Sociocultural Factors" |
| S107 | TX "Cultural adaptation" OR TX "Cultural adaptations" OR TX "Culturally adapted" |
| S108 | TX "cultural attunement" OR TX "cultural attunements" OR TX "culturally attuned" |
| S109 | TX "cultural adjustment" OR TX "cultural adjustments" OR TX “culturally adjusted |
| S110 | TX "culturally tailored" OR TX "cultural tailoring" |
| S111 | TX "culturally tailored" OR TX "cultural tailoring" |
| S112 | TX "Cultural modification" OR TX "Cultural modifications" OR TX "Culturally modified" |
| S113 | TX “contextual adaptation” OR TX “contextual adaptation” |
| S114 | TX “local adaptation” OR TX “local adaptations” |
| S115 | TX "Cultural consideration" OR TX "Cultural considerations" |
| S116 | TX "culturally suitable" OR TX "cultural suitability" |
| S117 | TX "culturally adequate" OR TX "cultural adequacy" |
| S118 | TX "culturally appropriate" OR TX "cultural appropriateness" |
| S119 | TX "Cultural influence" OR TX "Cultural influences" OR TX "Culturally influenced" |
| S120 | TI CALD OR AB CALD |
| S121 | TI "culturally diverse" OR AB "culturally diverse" OR TI "cultural diversity" OR AB "cultural diversity" |
| S122 | TI "linguistically diverse" OR AB "linguistically diverse" OR TI "linguistic diversity" OR AB "linguistic diversity" |
| S123 | TI "Cultural difference" OR AB "cultural difference" OR TI "Cultural differences" OR AB "Cultural differences" |
| S124 | TI "Culturally specific" OR AB "Culturally specific" OR TI "Cultural specificity" OR AB "Cultural specificity" OR TI "Cultural specificities" OR AB "Cultural specificities" OR TI "Cultural specific" OR AB "Cultural specific" |
| S125 | TI "Cultural identities" OR AB "Cultural identities" OR TI "Cultural identity" OR AB "Cultural identity" |
| S126 | TI "cultural background" OR AB "cultural background" OR TI "cultural backgrounds" OR AB "cultural backgrounds" |
| S127 | TI “culturally compared" OR AB “culturally compared" OR TI “cultural comparison” OR AB “cultural comparison” OR TI “cultural comparisons” OR AB “cultural comparisons” |
| S128 | TI “cultural group” OR AB “cultural group” OR TI “cultural groups” OR AB “cultural groups” |
| S129 | TI “cultural aspect” OR AB “cultural aspect” OR TI “cultural aspects” OR AB “cultural aspects” |
| S130 | TI "Cultural factor" OR AB "Cultural factor" OR TI "Cultural factors" OR AB "Cultural factors" |
| S131 | TI "culturally enhanced" OR AB "culturally enhanced" |
| S132 | TI "culturally grounded" OR AB "culturally grounded" |
| S133 | TI "Cultural equivalence" OR AB "Cultural equivalence" |
| S134 | TI "cultural fit" OR AB "cultural fit" |
| S135 | TI "cultural sensitivity" OR AB "cultural sensitivity" OR TI "cultural sensitive" OR AB "cultural sensitive" OR TI "culturally sensitive" OR AB "culturally sensitive" |
| S136 | TI "cultural awareness" OR AB "cultural awareness" |
| S137 | TI "Cultural knowledge" OR AB "Cultural knowledge" |
| S138 | TI “cultural understanding” OR AB “cultural understanding” |
| S139 | TI “cultural expertise” OR AB “cultural expertise” |
| S140 | TI “cultural skills” OR AB “cultural skills” |
| S141 | TI "Culturally informed" OR AB "Culturally informed" |
| S142 | TI "Culturally safe" OR AB "Culturally safe" OR TI "Cultural safety" OR AB "Cultural safety |
| S143 | TI "Culturally responsive" OR AB "Culturally responsive" OR TI "cultural responsiveness" OR AB "cultural responsiveness" |
| S144 | TI "Cultural focus" OR AB "Cultural focus" OR TI "Culturally focused" OR AB "Culturally focused" |
| S145 | TI "Culturally relevant" OR AB "Culturally relevant" OR TI "Cultural relevance" OR AB "Cultural relevance" |
| S146 | TI "Culturally congruent" OR AB "Culturally congruent" OR TI "Cultural congruence" OR AB "Cultural congruence" |
| S147 | TI "Culturally consistent" OR AB "Culturally consistent" OR TI "cultural consistency" OR AB "cultural consistency" |
| S148 | TI “multi cultur*” OR AB “multi cultur*” OR TI multicultur* OR AB multicultur* |
| S149 | TI “inter cultur*” OR AB “inter cultur*” OR TI “intercultur*” OR AB “intercultur |
| S150 | TI “cross cultur*” OR AB “cross cultur*” OR TI “crosscultur*” OR AB “crosscultur*” |
| S151 | TI sociocultur* OR AB sociocultur* OR TI "socio cultur*" OR AB "socio cultur*" |
| S152 | TI bicultur* OR AB bicultur* |
| S153 | TI bicultur* OR bicultur* |
| S154 | TI "ethnic bias" OR AB "ethnic bias" OR TI "ethnic biases" OR AB "ethnic biases" |
| S155 | TI "Racial bias" OR AB "Racial bias" OR TI "Racial biases" OR AB "Racial biases" |
| S156 | TI "ethnic disparity" OR AB "ethnic disparity" OR TI "ethnic disparities" OR AB "ethnic disparities" |
| S157 | TI "Racial disparity" OR AB "Racial disparity" OR TI "Racial disparities" OR AB "Racial disparities" |
| S158 | TI "ethnic diversity" OR AB "ethnic diversity" OR TI "ethnic diversities" OR AB "ethnic diversities" OR TI "ethnically diverse" OR AB "ethnically diverse" |
| S159 | TI “racial group” OR AB “racial group” OR TI "racial groups" OR AB "racial groups" |
| S160 | TI “ethnic group” OR AB “ethnic group” OR TI "ethnic groups" OR AB "ethnic groups" |
| S161 | TI “ethnic aspect” OR AB “ethnic aspect” OR TI "ethnic aspects" OR AB "ethnic aspects" |
| S162 | TI “racial aspect” OR AB “racial aspect” OR TI "racial aspects" OR AB "racial aspects" |
| S163 | TI racial OR TI race OR TI races OR TI ethnic OR TI ethnicity OR TI ethnicities OR TI culture OR TI cultures OR TI cultural OR TI minority OR TI minorities OR TI language OR TI linguistic |
| S164 | TI barriers N2 S163 |
| S165 | AB racial OR AB race OR AB races OR AB ethnic OR AB ethnicity OR AB ethnicities OR AB culture OR AB cultures OR AB cultural OR AB minority OR AB minorities OR AB language OR AB linguistic |
| S166 | AB barriers N2 S165 |
| S167 | TI racial OR TI race OR TI races OR TI ethnic OR TI ethnicity OR TI ethnicities OR TI culture OR TI cultures OR TI cultural OR TI minority OR TI minorities |
| S168 | AB racial OR AB race OR AB races OR AB ethnic OR AB ethnicity OR AB ethnicities OR AB culture OR AB cultures OR AB cultural OR AB minority OR AB minorities |
| S169 | DE "Communication Barriers" AND ( S167 OR S168 ) |
| S170 | DE "Health disparities" AND ( S167 OR S168 ) |
| S171 | ( TI "Health inequality" OR AB "Health inequality" ) AND ( S167 OR S168 ) |
| S172 | DE “Treatment compliance” AND ( S167 OR S168 ) |
| S173 | TI “culturally acceptable” OR AB “culturally acceptable” OR TI “cultural acceptability” OR AB “cultural acceptability” |
| S174 | TI "Cultural socialisation" OR AB "Cultural socialisation" OR TI "Cultural socialization" OR AB "Cultural socialization" |
| S175 | S96 OR S97 OR S98 OR S99 OR S100 OR S101 OR S102 OR S103 OR S104 OR S105 OR S106 OR S107 OR S108 OR S109 OR S110 OR S111 OR S112 OR S113 OR S114 OR S115 OR S116 OR S117 OR S118 OR S119 S120 OR S121 OR S122 OR S123 |
| S176 | S124 OR S125 OR S126 OR S127 OR S128 OR S129 OR S130 OR S131 OR S132 OR S133 OR S134 OR S135 OR S136 OR S137 OR S138 OR S139 OR S140 OR S141 OR S142 OR S143 OR S144 OR S145 OR S146 OR S147 OR S148 OR S149 OR S150 OR S151 OR S152 OR S153 |
| S177 | S154 OR S155 OR S156 OR S157 OR S158 OR S159 OR S160 OR S161 OR S162 OR S164 OR S166 OR S169 OR S170 OR S171 OR S172 OR S173 OR S174 |
| S178 | S175 OR S176 OR S177 |
| S179 | TI Africa OR TI African OR TI Africans |
| S180 | TI Asia OR TI Asian OR TI Asians |
| S181 | TI “Middle America” OR TI “Middle American” OR TI “Middle Americans” |
| S182 | TI “South America” OR TI “South American” OR TI “South Americans” |
| S183 | TI “Central America” OR TI “Central American” OR TI “Central Americans” |
| S184 | TI Arab OR TI Arabs |
| S185 | TI “Middle East” OR TI “Middle Eastern” |
| S186 | TI Afghanistan OR TI Afghan OR TI Afghans |
| S187 | TI Albania OR TI Albanian OR TI Albanians |
| S188 | TI Algeria OR TI Algerian OR TI Algerians |
| S189 | TI Angolans OR TI Angolan OR TI Angolans |
| S190 | TI Antilles |
| S191 | TI Antigua OR TI Antiguan OR TI Antiguans OR TI Barbuda OR TI Barbadian OR TI Barbadians |
| S192 | TI Argentina OR TI Argentinian OR TI Argentine OR TI Argentines OR TI Argentinean OR TI Argentinians OR TI Argentineans |
| S193 | TI Armenia OR TI Armenian OR TI Armenians |
| S194 | TI Aruba OR TI Aruban OR TI Arubans |
| S195 | TI Azerbaijan OR TI Azerbaijani OR TI Azerbaijanis OR TI Azeri |
| S196 | TI Bahamas |
| S197 | TI Bahrain OR TI Bahraini OR TI Bahrainis |
| S198 | TI Bajun OR TI Bajuni OR TI Bajunis |
| S199 | TI Bangladesh OR TI Bangladeshi OR TI Bangladeshis |
| S200 | TI Barbados |
| S201 | TI Belize OR TI Belizean OR TI Belizeans |
| S202 | TI Benin OR TI Beninese |
| S203 | TI Bermuda |
| S204 | TI Byelarus OR TI Byelorussian OR TI Belarus OR TI Belorussian OR TI Belorussia OR TI Belarussian OR TI Byelorussions OR TI Belorussians OR TI Belarussions |
| S205 | TI Bhutan OR TI Bhutanese |
| S206 | TI Bolivia OR TI Bolivian OR TI Bolivians |
| S207 | TI Bosnia OR TI Bosnian OR TI Bosnians |
| S208 | TI Herzegovina OR TI Hercegovina OR TI Herzegovinian OR TI Herzegovinians |
| S209 | TI Botswana OR TI Batswana |
| S210 | TI Brasil OR TI Brazil OR TI Brazilian OR TI Brazils |
| S211 | TI Brunei OR TI Darussalam |
| S212 | TI Bulgaria OR TI Bulgarian OR TI Bulgarians |
| S213 | TI Burkina OR TI Burkinabe OR TI Faso OR TI Fasso OR TI Volta |
| S214 | TI Burundi OR TI Burundian OR TI Burundians OR TI Urund |
| S215 | TI Caledonia OR TI Caledonian OR TI Caledonians |
| S216 | TI Cambodia OR TI Cambodian OR TI Cambodians OR TI Khmer OR TI Kampuchea |
| S217 | TI Cameroon OR TI Cameroons OR TI Cameroonian OR TI Cameron OR TI Camerons |
| S218 | TI "Cape Verde" OR TI "Cape Verdian" OR TI "Cabo Verde" OR TI "Cabo Verdian" OR TI "Cabo Verdians" OR TI "Cape Verdians" OR TI "Cape Verdeans" OR TI "Cape Verdean" |
| S219 | TI Caribbean OR TI Caribbeans |
| S220 | TI Chad OR TI Chadian OR TI Chadians |
| S221 | TI Chile OR TI Chilean OR TI Chileans |
| S222 | TI China OR TI Chinese |
| S223 | TI Colombia OR TI Colombian OR TI Colombians |
| S224 | TI Comoros OR TI Comoro OR TI Comores OR TI Comoran OR TI Comorans |
| S225 | TI Mayotte OR TI Mahorais |
| S226 | TI Congo OR TI Congolese OR TI Zaire |
| S227 | TI "Cook Islands" |
| S228 | TI Rica OR TI Rican OR TI Ricans |
| S229 | TI d'Ivoire OR TI Ivory OR TI Ivoirian OR TI Ivoirians |
| S230 | TI Cuba OR TI Cuban OR TI Cubans |
| S231 | TI Curacao OR TI Curacaoan OR TI Curacaoans |
| S232 | TI Cyprus OR TI Cyprian OR TI Cyprians |
| S233 | TI Djibouti |
| S234 | TI Dominica OR TI Dominican OR TI Dominicans |
| S235 | TI Ecuador OR TI Ecuadorian OR TI Ecuadorians |
| S236 | TI Egypt OR TI Egyptian OR TI Egyptians |
| S237 | TI Salvador OR TI Salvadorian OR TI Salvadorians |
| S238 | TI Emirates OR TI Emirati |
| S239 | TI Eritrea OR TI Eritrean OR TI Eritreans |
| S240 | TI Eswatini OR TI Swaziland OR TI Swazi OR TI Swazis |
| S241 | TI Ethiopia OR TI Ethiopian OR TI Ethiopians |
| S242 | TI Faroe |
| S243 | TI Fiji OR TI Fijian |
| S244 | TI Gabon OR TI Gabonese |
| S245 | TI Gambia OR TI Gambian OR TI Gambians |
| S246 | TI Georgia OR TI Georgian OR TI Georgians |
| S247 | TI Ghana OR TI Ghanaian OR TI Ghanaians |
| S248 | TI Gibraltar OR TI Gibraltarian OR TI Gibraltarians |
| S249 | TI Grenada OR TI Grenadian OR TI Grenadians |
| S250 | TI Guam OR TI Guamanian OR TI Guamanians OR TI Chamorro |
| S251 | TI Guatemala OR TI Guatemalan OR TI Guatemalans |
| S252 | TI Guinea OR TI Guinean OR TI Guineans |
| S253 | TI Guiana OR TI Guyana OR TI Guyanese OR TI Guianan OR TI Guianans OR TI Guianese |
| S254 | TI Haiti OR TI Haitian OR TI Haitians |
| S255 | TI Honduras OR TI Honduran OR TI Hondurans |
| S256 | TI "Hong Kong" OR TI "Hong Kongese" |
| S257 | TI India OR TI Indian OR TI Indians |
| S258 | TI Indonesia OR TI Indonesian OR TI Indonesians |
| S259 | TI Iran OR TI Iranian OR TI Iranians |
| S260 | TI Persia OR TI Persian OR TI Persians |
| S261 | TI Iraq OR TI Iraqi |
| S262 | TI Israel OR TI Israeli |
| S263 | TI Jamaica OR TI Jamaican OR TI Jamaicans |
| S264 | TI Japan OR TI Japanese |
| S265 | TI Jordan OR TI Jordanian OR TI Jordanians |
| S266 | TI Kazakhstan OR TI Kazakh OR TI Kazakhs |
| S267 | TI Kenya OR TI Kenyan OR TI Kenyans |
| S268 | TI Kiribati OR TI Kiribatian OR TI Kiribatians |
| S269 | TI Korea OR TI Korean OR TI Koreans |
| S270 | TI Kosovo OR TI Kosovar |
| S271 | TI Kuwait OR TI Kuwaiti OR TI Kuwaitis |
| S272 | TI Kyrgyzstan OR TI Kirghizia OR TI Kyrgyz OR TI Kirghiz OR TI Kirgizstan OR TI Kyrgyzstani OR TI Kyrgyzstanis |
| S273 | TI Lao OR TI Laos OR TI Laotian OR TI Laotians |
| S274 | TI Latin OR TI Latino OR TI Latinos |
| S275 | TI Lebanon OR TI Lebanese |
| S276 | TI Lesotho OR TI Lesothan OR TI Lesothans OR TI Basutoland OR TI Basotho |
| S277 | TI Liberia OR TI Liberian OR TI Liberians |
| S278 | TI Libya OR TI Libyan OR TI Libyans |
| S279 | TI Macao OR TI Macau OR TI Macanese |
| S280 | TI Macedonia OR TI Macedonian OR TI Macedonians |
| S281 | TI Madagasca OR TI Madagascan OR TI Madagascans OR TI Malagasy |
| S282 | TI Malawi OR TI Malawian OR TI Nyasaland |
| S283 | TI Malaysia OR TI Malaysian OR TI Malaysians OR TI Malaya OR TI Malay OR TI Sabah OR TI Sarawak |
| S284 | TI Maldives OR TI Maldivian OR TI Maldivians |
| S285 | TI Mali OR TI Malian OR TI Malians |
| S286 | TI Mariana |
| S287 | TI Marshall OR TI Marshallese |
| S288 | TI Mauritania OR TI Mauritanian OR TI Mauritanians |
| S289 | TI Mauritius OR TI Mauritian OR TI Mauritians OR TI Agalega |
| S290 | TI Mexico OR TI Mexican OR TI Mexicans |
| S291 | TI Micronesia OR TI Micronesian OR TI Micronesians OR TI Mariana |
| S292 | TI moldova OR TI Moldovian OR TI Moldovia OR TI Moldovan OR TI Moldovans OR TI Moldovians |
| S293 | TI Monaco OR TI Monacan OR TI Monacans |
| S294 | TI Mongolia OR TI Mongolian OR TI Mongolians |
| S295 | TI Montenegro OR TI Montenegrin OR TI Montenegrins |
| S296 | TI Montserrat |
| S297 | TI Morocco OR TI Moroccan OR TI Moroccans OR TI Ifni |
| S298 | TI Mozambique OR TI Mozambican OR TI Mozambicans |
| S299 | TI myanmar OR TI Myanmarese OR TI Burma OR TI Burmese |
| S300 | TI Namibia OR TI Namibian OR TI Namibians |
| S301 | TI Nauru OR TI Nauruan OR TI Nauruans |
| S302 | TI Nepal OR TI Nepalese |
| S303 | TI Nicaragua OR TI Nicaraguan OR TI Nicaraguans |
| S304 | TI Niger |
| S305 | TI Nigeria OR TI Nigerian OR TI Nigerians |
| S306 | TI Niue |
| S307 | TI Oman OR TI Omani OR TI Omanis OR TI Muscat |
| S308 | TI Pakistan OR TI Pakistani OR TI Pakistanis |
| S309 | TI Palau OR TI Palauan OR TI Palauans |
| S310 | TI Palestina OR TI Palestinian OR TI Palestinians OR TI Gaza OR TI West-Bank OR TI "West Bank" |
| S311 | TI Panama OR TI Panamanian OR TI Panamanians |
| S312 | TI Paraguay OR TI Paraguayan OR TI Paraguayans |
| S313 | TI Peru OR TI Peruvian OR TI Peruvians |
| S314 | TI Philippines OR TI Philipines OR TI Phillipines OR TI Phillippines OR TI Filipino OR TI Filipinos OR TI Philippino OR TI Philippinos |
| S315 | TI Polynesia OR TI Polynesian OR TI Polynesians |
| S316 | TI "Puerto Rico" OR TI "Puerto Rican" OR TI "Puerto Ricans" |
| S317 | TI Qatar OR TI Qatari OR TI Qataris |
| S318 | TI Rhodesia OR TI Rhodesian OR TI Rhodesians |
| S319 | TI Romania OR TI Roumania OR TI Romanian OR TI Romanians OR TI Rumania |
| S320 | TI Russia OR TI Russian OR TI Russians |
| S321 | TI Rwanda OR TI Ruanda OR TI Rwandan OR TI Rwandans |
| S322 | TI Kitts OR TI Nevis |
| S323 | TI Lucia OR TI Lucian OR TI Lucians |
| S324 | TI Vincent OR TI Grenadines |
| S325 | TI "Saint Helena" |
| S326 | TI Samoa OR TI Samoan OR TI Samoans |
| S327 | TI "San Marino" OR TI "San Marinese" |
| S328 | TI "Sao Tome" OR TI "Sao Tomean" OR TI "Sao Tomeans" OR TI Principe |
| S329 | TI Saudi OR TI Arabian OR TI Arabians OR TI Arabia |
| S330 | TI Senegal OR TI Senegalese |
| S331 | TI Serbia OR TI Serbian OR TI Serbians |
| S332 | TI seychelles OR TI Seychellois |
| S333 | TI Sierra OR TI Leone OR TI Leonean OR TI Leoneans |
| S334 | TI Singapore OR TI Singaporean OR TI Singaporeans |
| S335 | TI Slovakia OR TI Slovakian OR TI Slovakians |
| S336 | TI Slovenia OR TI Slovenian OR TI Slovenians |
| S337 | TI Solomon |
| S338 | TI Somalia OR TI Somali OR TI Somalis OR TI Somaliland OR TI Somalian OR TI Somalians |
| S339 | TI "Sri Lanka" OR TI "Sri Lankan" OR TI "Sri Lankans" OR TI Ceylon |
| S340 | TI Sudan OR TI Sudanese |
| S341 | TI Suriname OR TI Surinam OR TI Surinen |
| S342 | TI Syria OR TI Syrian OR TI Syrians |
| S343 | TI Taiwan OR TI Taiwanese |
| S344 | TI Tajikistan OR TI Tadzhikistan OR TI Tadjikistan OR TI Tadzhik OR TI Tadzhiks OR TI Tajik OR TI Tajiks |
| S345 | TI Tanzania OR TI Tanzanian OR TI Tanzanians |
| S346 | TI Thailand OR TI Thai OR TI Thais |
| S347 | TI Timor OR TI Timur OR TI Atoni OR TI Atonis OR TI Leste |
| S348 | TI Tobagonian OR TI Tobagonians |
| S349 | TI Togo OR TI Togolese |
| S350 | TI Tokelau |
| S351 | TI Tonga OR TI Tongan OR TI Tongans |
| S352 | TI Tobago OR TI Trinidad OR TI Trinidadian OR TI Trinidadians |
| S353 | TI Tunisia OR TI Tunisian OR TI Tunisians |
| S354 | TI Turkey OR TI Turkish |
| S355 | TI Turkmenistan OR TI Turkmen OR TI Turkmenistani OR TI Turkmenistanis |
| S356 | TI Tuvalu OR TI Tuvaluan OR TI Tuvaluans |
| S357 | TI Uganda OR TI Ugandan OR TI Ugandans |
| S358 | TI Ukraine OR TI Ukrainian OR TI Ukrainians |
| S359 | TI Uruguay OR TI Uruguayan OR TI Uruguayans |
| S360 | TI USSR OR TI Soviet OR TI Soviets |
| S361 | TI Uzbekistan OR TI Uzbek OR TI Uzbeks |
| S362 | TI Vanuatu OR TI Vanuatuan OR TI Vanuatuans |
| S363 | TI Venezuela OR TI Venezuelan OR TI Venezuelans |
| S364 | TI Vietnam OR TI "Viet Nam" OR TI Vietnamese |
| S365 | TI Wallis |
| S366 | TI Yemen OR TI Yemeni OR TI Yemenis |
| S367 | TI Yugoslavia OR TI Yugoslavian OR TI Yugoslavians OR TI Yugoslav OR TI Yugoslavs OR TI Jugoslavia |
| S368 | TI Zambia OR TI Zambian OR TI Zambians |
| S369 | TI Zimbabwe OR TI Zimbabwean OR TI Zimbabweans |
| S370 | S179 OR S180 OR S181 OR S182 OR S183 OR S184 OR S185 OR S186 OR S187 OR S188 OR S189 OR S190 OR S191 OR S192 OR S193 OR S194 OR S195 OR S196 OR S197 OR S198 OR S199 OR S200 OR S201 OR S202 OR S203 OR S204 OR S205 OR S206 OR S207 OR S208 OR S209 OR S210 OR S211 OR S212 OR S213 OR S214 OR S215 OR S216 OR S217 OR S218 OR S219 OR S220 OR S221 OR S222 OR S223 OR S224 OR S225 OR S226 OR S227 OR S228 OR S229 OR S230 OR S231 OR S232 OR S233 OR S234 OR S235 OR S236 OR S237 OR S238 OR S239 OR S240 OR S241 OR S242 OR S243 OR S244 OR S245 OR S246 OR S247 OR S248 OR S249 OR S250 OR S251 OR S252 OR S253 OR S254 OR S255 OR S256 OR S257 OR S258 OR S259 OR S260 OR S261 OR S262 OR S263 OR S264 OR S265 OR S266 OR S267 OR S268 OR S269 OR S270 OR S271 OR S272 OR S273 OR S274 OR S275 OR S276 OR S277 OR S278 OR S279 OR S280 OR S281 OR S282 OR S283 OR S284 OR S285 OR S286 OR S287 OR S288 OR S289 OR S290 OR S291 OR S292 OR S293 OR S294 OR S295 OR S296 OR S297 OR S298 OR S299 OR S300 OR S301 OR S302 OR S303 OR S304 OR S305 OR S306 OR S307 OR S308 OR S309 OR S310 OR S311 OR S312 OR S313 OR S314 OR S315 OR S316 OR S317 OR S318 OR S319 OR S320 OR S321 OR S322 OR S323 OR S324 OR S325 OR S326 OR S327 OR S328 OR S329 OR S330 OR S331 OR S332 OR S333 OR S334 OR S335 OR S336 OR S337 OR S338 OR S339 OR S340 OR S341 OR S342 OR S343 OR S344 OR S345 OR S346 OR S347 OR S348 OR S349 OR S350 OR S351 OR S352 OR S353 OR S354 OR S355 OR S356 OR S357 OR S358 OR S359 OR S360 OR S361 OR S362 OR S363 OR S364 OR S365 OR S366 OR S367 OR S368 OR S369 |
| S371 | TI LAMIC OR AB LAMIC OR TI LAMICs OR AB LAMICs |
| S372 | TI LMIC OR AB LMIC OR TI LMICs OR AB LMICs |
| S373 | TI "LAMI Country" OR AB "LAMI Country" OR TI "LAMI Countries" OR AB "LAMI Countries" |
| S374 | TI "LMI Country" OR AB "LMI Country" OR TI "LMI Countries" OR AB "LMI Countries" |
| S375 | TI "third world" OR AB "third world" |
| S376 | ( TI developing OR TI "less developed" OR TI "under developed" OR underdeveloped OR TI "least developed" OR TI crisis OR TI war OR TI "middle income" OR TI "low income" OR TI poor ) N2 ( TI country OR TI countries OR TI nation OR TI nations OR TI area OR TI areas OR TI economy OR TI economies OR TI population OR TI populations ) |
| S377 | DE "Developing Countries" |
| S378 | DE "Human Migration" |
| S379 | DE "Refugees" |
| S380 | DE "Immigration" |
| S381 | TI BME OR TI BEM OR TI BAME OR TI afro |
| S382 | TI "black and minority ethnic" OR TI "black and ethnic minority" OR TI "black, Asian and minority ethnic" |
| S383 | TI Kurdish OR TI Kurd OR TI Kurds OR TI Kurdistan |
| S384 | TI Amerindian OR TI Amerindians |
| S385 | TI Hispanic OR TI Hispanics |
| S386 | TI Yezidi OR TI Yezidis OR TI Yazidi OR TI Yazidis |
| S387 | TI Inuit OR TI Inuits OR TI "first nations" OR TI indigenous |
| S388 | TI Eskimo OR TI Eskimos |
| S389 | TI Kalaallit OR TI Kalaallits |
| S390 | TI Romany OR TI Romanies |
| S391 | TI Gypsies OR TI Gipsies OR TI Gypsy OR TI Gipsy |
| S392 | TI Aborigine OR TI Aborigines |
| S393 | TI Refugee OR TI refugees |
| S394 | TI "Asylum seeker" OR TI "Asylum seekers" |
| S395 | TI Migrant OR TI Migrants |
| S396 | TI immigrant OR TI immigrants |
| S397 | TI emigrant OR TI emigrants |
| S398 | TI (person OR persons OR people OR group OR groups) |
| S399 | (TI displaced) N S398 |
| S400 | S371 OR S372 OR S373 OR S374 OR S375 OR S376 OR S377 OR S378 OR S379 OR S380 |
| S401 | S381 OR S382 OR S383 OR S384 OR S385 OR S386 OR S387 OR S388 OR S389 OR S390 OR S391 OR S392 |
| S402 | S393 OR S394 OR S395 OR S396 OR S397 OR S399 |
| S403 | S400 OR S401 OR S402 |
| S404 | S178 OR S370 OR S403 |
| S405 | DE "Mental Disorders" |
| S406 | DE "Chronic Mental Illness" |
| S407 | DE "Behavior Disorders" |
| S408 | DE "Emotional Adjustment" OR DE "Emotional Disturbances" |
| S409 | DE "Emotional Disturbances" |
| S410 | DE "Psychiatric Patients" |
| S411 | DE "Psychiatric Symptoms" |
| S412 | DE "Psychopathology" |
| S413 | DE "Thought Disturbances" |
| S414 | DE "Psychological Stress" |
| S415 | DE "Psychological Endurance" |
| S416 | DE "Behavior Problems" |
| S417 | DE "Mental Health" |
| S418 | TI “mental distress“ OR AB “mental distress“ OR TI “mental stress” OR AB “mental stress” OR TI “mentally stressed” OR AB “mentally stressed” |
| S419 | TI “mental issue” OR AB “mental issue” OR TI “mental issues” OR AB “mental issues” |
| S420 | TI “mental disturbance” OR AB “mental disturbance” OR TI “mental disturbances” OR AB “mental disturbances” OR TI “mentally disturbed” OR AB “mentally disturbed” |
| S421 | TI “mental problem” OR AB “mental problem” OR TI “mental problems” OR AB “mental problems” |
| S422 | TI “mental disorder” OR AB “mental disorder” OR TI “mental disorders” OR AB “mental disorders” |
| S423 | TI “mental disease” OR AB “mental disease” OR TI “mental diseases” OR AB “mental diseases” |
| S424 | TI “mental illness” OR AB “mental illness” OR TI “mental illnesses” OR AB “mental illnesses” OR TI “mentally ill” OR AB “mentally ill” |
| S425 | TI “psychiatric disorder” OR AB “psychiatric disorder” OR TI “psychiatric disorders” OR AB “psychiatric disorders” |
| S426 | TI “psychiatric disease” OR AB “psychiatric disease” OR TI “psychiatric diseases” OR AB “psychiatric diseases” |
| S427 | TI “psychiatric illness” OR AB “psychiatric illness” OR TI “psychiatric illnesses” OR AB “psychiatric illnesses” |
| S428 | TI “psychological illness” OR AB “psychological illness” OR TI “psychological illnesses” OR AB “psychological illnesses” OR TI “psychologically ill” OR AB “psychologically ill” OR TI “psychically ill” OR AB “psychically ill” |
| S429 | TI “psychological disorder” OR AB “psychological disorder” OR TI “psychological disorders” OR AB “psychological disorders” |
| S430 | TI “psychological disease” OR AB “psychological disease” OR TI “psychological diseases” OR AB “psychological diseases” |
| S431 | TI “emotional strains” OR AB “emotional strains” OR TI “emotional strain” OR AB “emotional strain” OR TI “emotionally strained” OR AB “emotionally strained” |
| S432 | TI “emotional stress” OR AB “emotional stress” OR TI “emotional distress” OR AB “emotional distress” OR TI “emotionally stressed” OR AB “emotionally stressed” |
| S433 | TI “emotional disturbance” OR AB “emotional disturbance” OR TI “emotional disturbances” OR AB “emotional disturbances” OR TI “emotionally disturbed” OR AB “emotionally disturbed” |
| S434 | TI “emotional damage” OR AB “emotional damage” |
| S435 | TI “psychically disturbed” OR AB “psychically disturbed” |
| S436 | TI “psychic strain” OR AB “psychic strain” OR TI “psychic strains” OR AB “psychic strains” OR TI “psychically strained” OR AB “psychically strained” |
| S437 | TI “psychic distress” OR AB “psychic distress” OR TI “psychic stress” OR AB “psychic stress” OR TI “psychically stressed” OR AB “psychically stressed” |
| S438 | TI “Psychological distress” OR AB “Psychological distress” OR TI “Psychological stress” OR AB “Psychological stress” OR TI “Psychologically stressed” OR AB “Psychologically stressed” |
| S439 | TI “Psychological strain” OR AB “Psychological strain” OR TI “Psychological strains” OR AB “Psychological strains” OR TI “Psychologically strained” OR AB “Psychologically strained” |
| S440 | TI “mental ill-health” OR AB “mental ill-health” OR TI “psychological ill-health” OR AB “psychological ill-health” |
| S441 | TI “mental health” OR AB “mental health” OR TI “mental health” OR AB “mental health” |
| S442 | TI “mental well being” OR AB “mental well being” OR TI “mental wellbeing” OR AB “mental wellbeing” |
| S443 | TI “psychological well being” OR AB “psychological well being” OR TI “psychological wellbeing” OR AB “psychological wellbeing” |
| S444 | S405 OR S406 OR S407 OR S408 OR S409 OR S410 OR S411 OR S412 OR S413 OR S414 OR S415 OR S416 OR S417 OR S418 OR S419 OR S420 OR S421 OR S422 OR S423 OR S424 OR S425 OR S426 OR S427 OR S428 OR S429 OR S430 OR S431 OR S432 OR S433 OR S434 OR S435 OR S436 OR S437 OR S438 OR S439 OR S440 OR S441 OR S442 OR S443 |
| S445 | DE "Adjustment Disorders" |
| S446 | DE "Affective Disorders" |
| S447 | DE "Bipolar Disorder" |
| S448 | DE "Cyclothymic Personality" |
| S449 | DE "Disruptive Mood Dysregulation Disorder" |
| S450 | DE "Major Depression" |
| S451 | DE "Dysthymic Disorder" |
| S452 | DE "Mania" |
| S453 | DE "Hypomania" |
| S454 | DE "Seasonal Affective Disorder" |
| S455 | DE “anhedonia” |
| S456 | TI "Bipolar Disorder" OR AB "Bipolar Disorder" OR TI "Bipolar Disorders" OR AB "Bipolar Disorders" |
| S457 | TI "Mood Disorder" OR AB "Mood Disorder" OR TI "Mood Disorders" OR AB "Mood Disorders" |
| S458 | TI "Cyclothymic Disorder" OR AB "Cyclothymic Disorder" OR TI "Cyclothymic Disorders" OR AB "Cyclothymic Disorders" OR TI "premenstrual dysphoric disorder" OR AB "premenstrual dysphoric disorder" OR TI "premenstrual dysphoric disorders" OR AB "premenstrual dysphoric disorders" |
| S459 | TI "affective Disorder" OR AB "affective Disorder" OR TI "affective Disorders" OR AB "affective Disorders" |
| S460 | TI depression OR AB depression OR TI depressions OR AB depressions OR TI depressed OR AB depressed OR TI depressive OR AB depressive |
| S461 | TI dysthymic OR AB dysthymic OR TI dysthymia OR AB dysthymia OR TI melancholy OR AB melancholy OR TI melancholic OR AB melancholic OR TI melancholia OR AB melancholia |
| S462 | S445 OR S446 OR S447 OR S448 OR S449 OR S450 OR S451 OR S452 OR S453 OR S454 OR S455 OR S456 OR S457 OR S458 OR S459 OR S460 OR S461 |
| S463 | DE "Anxiety Disorders" |
| S464 | DE "Acute Stress Disorder" |
| S465 | DE "Generalized Anxiety Disorder" |
| S466 | DE "Obsessive Compulsive Disorder" |
| S467 | DE "Panic Disorder" |
| S468 | DE "Phobias" |
| S469 | DE "Acrophobia" |
| S470 | DE "Agoraphobia" |
| S471 | DE "Claustrophobia" |
| S472 | DE "Social Phobia" |
| S473 | DE "Separation Anxiety Disorder" |
| S474 | DE “anxiety” |
| S475 | DE “Fear” |
| S476 | DE “Panic” |
| S477 | DE "Panic Attack" |
| S478 | TI agoraphobia OR AB agoraphobia |
| S479 | TI Anxiety OR AB Anxiety |
| S480 | TI Neurotic OR AB Neurotic |
| S481 | TI "Obsessive-Compulsive Disorder" OR AB "Obsessive-Compulsive Disorder" OR TI "Obsessive-Compulsive Disorders" OR AB "Obsessive-Compulsive Disorders" |
| S482 | TI phobic OR AB phobic OR TI phobia OR AB phobia |
| S483 | TI panic OR AB panic OR TI "selective mutism" OR AB "selective mutism" |
| S484 | S463 OR S464 OR S465 OR S466 OR S467 OR S468 OR S469 OR S470 OR S471 OR S472 OR S473 OR S474 OR S475 OR S476 OR S477 OR S478 OR S479 OR S480 OR S481 OR S482 OR S483 |
| S485 | DE "Post-Traumatic Stress" |
| S486 | DE "Posttraumatic Stress Disorder" |
| S487 | DE "Complex PTSD" |
| S488 | DE "Emotional Trauma" |
| S489 | TI trauma OR AB trauma OR TI traumatised OR AB traumatised OR TI traumatized OR AB traumatized |
| S490 | TI PTSD OR AB PTSD OR TI "stress disorder" OR AB "stress disorder" OR TI "stress disorders" OR AB "stress disorders" |
| S491 | TI "post traumatic" OR AB "post traumatic" OR TI posttraumatic OR AB posttraumatic OR TI traumatic OR AB traumatic |
| S492 | TI "Adjustment Disorders" OR AB "Adjustment Disorders" OR TI "Adjustment Disorder" OR AB "Adjustment Disorder" |
| S493 | S485 OR S486 OR S487 OR S488 OR S489 OR S490 OR S491 OR S492 |
| S494 | DE "Autism Spectrum Disorders" |
| S495 | DE "Dissociative Disorders" |
| S496 | DE "Depersonalization" |
| S497 | DE "Depersonalization/Derealization Disorder" |
| S498 | DE "Dissociative Identity Disorder" |
| S499 | DE "Dissociation" |
| S500 | TI "Dissociative Disorder" OR AB "Dissociative Disorder" OR TI "Dissociative Disorders" OR AB "Dissociative Disorders" |
| S501 | TI Depersonalisation OR AB Depersonalisation OR TI Depersonalization OR AB Depersonalization |
| S502 | TI Dissociation OR AB Dissociation |
| S503 | S494 OR S495 OR S496 OR S497 OR S498 OR S499 OR S500 OR S501 OR S502 |
| S504 | DE "Impulse Control Disorders" |
| S505 | DE "Impulsiveness" |
| S506 | DE "Kleptomania" |
| S507 | DE "Pathological Gambling" |
| S508 | DE "Trichotillomania" |
| S509 | DE "Hoarding Disorder" |
| S510 | DE "Hoarding Behavior" |
| S511 | TI "Impulse Control Disorder" OR AB "Impulse Control Disorder" OR TI "Impulse Control Disorders" OR AB "Impulse Control Disorders” |
| S512 | TI "hoarding behavior" OR AB "hoarding behavior" OR TI "hoarding disorder" OR AB "hoarding disorder" |
| S513 | TI gambling OR AB gambling |
| S514 | TI Trichotillomania OR AB Trichotillomania |
| S515 | S504 OR S505 OR S506 OR S507 OR S508 OR S509 OR S510 OR S511 OR S512 OR S513 OR S514 |
| S516 | DE "Eating Disorders" |
| S517 | DE "Anorexia Nervosa" |
| S518 | DE "Binge Eating Disorder" |
| S519 | DE "Bulimia" |
| S520 | DE "Purging (Eating Disorders)“ |
| S521 | DE "Binge Eating" |
| S522 | TI "Eating Disorder" OR AB "Eating Disorder" OR TI "Eating Disorders" OR AB "Eating Disorders" |
| S523 | TI Bulimia OR AB Bulimia |
| S524 | TI Anorexia OR AB Anorexia |
| S525 | TI "Binge Eating" OR AB "Binge Eating" |
| S526 | S516 OR S517 OR S518 OR S519 OR S520 OR S521 OR S522 OR S523 OR S524 OR S525 |
| S527 | DE "Personality Disorders" |
| S528 | DE "Borderline States" |
| S529 | DE "Obsessive Compulsive Personality Disorder" |
| S530 | DE "Histrionic Personality Disorder" |
| S531 | DE "Neurosis" |
| S532 | DE "Antisocial Personality Disorder" |
| S533 | DE "Avoidant Personality Disorder" |
| S534 | DE "Borderline Personality Disorder" |
| S535 | DE "Dependent Personality Disorder" |
| S536 | DE "Narcissistic Personality Disorder" |
| S537 | DE "Paranoid Personality Disorder" |
| S538 | DE "Passive Aggressive Personality Disorder" |
| S539 | DE "Schizoid Personality Disorder" |
| S540 | DE "Schizotypal Personality Disorder" |
| S541 | TI "Personality Disorder" OR AB "Personality Disorder" OR TI "Personality Disorders" OR AB "Personality Disorders" |
| S542 | TI Borderline OR AB Borderline |
| S543 | TI histrionic OR AB histrionic |
| S544 | TI Paranoid OR AB Paranoid |
| S545 | TI schizoid OR AB schizoid |
| S546 | TI Schizotypal OR AB Schizotypal |
| S547 | TI narcism OR AB narcism |
| S548 | DE "Psychosis" |
| S549 | DE "Affective Psychosis" |
| S550 | DE "Schizophrenia" |
| S551 | DE "Paranoia (Psychosis)" |
| S552 | DE "Paranoid Schizophrenia" |
| S553 | DE "Schizoaffective Disorder" |
| S554 | TI Schizophrenia OR AB Schizophrenia |
| S555 | TI Psychotic OR AB Psychotic |
| S556 | TI psychosis OR AB psychosis OR TI psychoses OR AB psychoses |
| S557 | TI schizoaffective OR AB schizoaffective |
| S558 | TI schizophrenic OR AB schizophrenic |
| S559 | TI "Multiple Personality" OR AB "Multiple Personality" OR TI "Multiple Personalities" OR AB "Multiple Personalities" |
| S560 | S527 OR S528 OR S529 OR S530 OR S531 OR S532 OR S533 OR S534 OR S535 OR S536 OR S537 OR S538 OR S539 OR S540 OR S541 OR S542 OR S543 OR S544 OR S545 OR S546 OR S547 OR S548 OR S549 OR S550 OR S551 OR S552 OR S553 OR S554 OR S555 OR S556 OR S557 OR S558 OR S559 |
| S561 | DE "Sleep Disorders" |
| S562 | DE "Hypersomnia" |
| S563 | DE "Insomnia" |
| S564 | DE "Parasomnias" |
| S565 | DE "Sleep Apnea" |
| S566 | DE "Sleep Deprivation" |
| S567 | DE “sleep” |
| S568 | TI "Sleep Wake Disorder" OR AB "Sleep Wake Disorder" OR TI "Sleep Wake Disorders" OR AB "Sleep Wake Disorders" |
| S569 | TI "Sleep Disorder" OR AB "Sleep Disorder" OR TI "Sleep Disorders" OR AB "Sleep Disorders" |
| S570 | TI "Sleeping Disorder" OR AB "Sleeping Disorder" OR TI "Sleeping Disorders" OR AB "Sleeping Disorders" |
| S571 | TI "Sleeping problem" OR AB "Sleeping problem" OR TI "Sleeping problems" OR AB "Sleeping problems" |
| S572 | TI "sleep problem" OR AB "sleep problem" OR TI "sleep problems" OR AB "sleep problems" |
| S573 | TI Dyssomnia OR AB Dyssomnia |
| S574 | TI Parasomnia OR AB Parasomnia |
| S575 | TI insomnia OR AB insomnia |
| S576 | TI "restless legs" OR AB "restless legs" |
| S577 | S561 OR S562 OR S563 OR S564 OR S565 OR S566 OR S567 OR S568 OR S569 OR S570 OR S571 OR S572 OR S573 OR S574 OR S575 OR S576 |
| S578 | DE "Toxic Disorders" |
| S579 | DE "Substance Use Disorder" |
| S580 | DE "Alcohol Intoxication" |
| S581 | DE "Alcoholism" |
| S582 | TI "Substance-Related Disorder" OR AB "Substance-Related Disorder" OR TI "Substance-Related Disorders" OR AB "Substance-Related Disorders" |
| S583 | TI "Alcohol-Related Disorder" OR AB "Alcohol-Related Disorder" OR TI "Alcohol-Related Disorders" OR AB "Alcohol-Related Disorders" |
| S584 | TI alcoholism OR AB alcoholism |
| S585 | TI "alcohol dependence" OR AB "alcohol dependence" |
| S586 | TI "alcohol dependency" OR AB "alcohol dependency" |
| S587 | TI "alcohol abuse" OR AB "alcohol abuse" |
| S588 | TI "substance abuse" OR AB "substance abuse" |
| S589 | TI "substance dependence" OR AB "substance dependence" |
| S590 | TI "substance dependency" OR AB "substance dependency" |
| S591 | TI addiction OR AB addiction |
| S592 | S578 OR S579 OR S580 OR S581 OR S582 OR S583 OR S584 OR S585 OR S586 OR S587 OR S588 OR S589 OR S590 OR S591 |
| S593 | TI "somatic symptom disorder" OR AB "somatic symptom disorder" OR TI "somatic symptom disorders" OR AB "somatic symptom disorders" |
| S594 | TI somatization OR AB somatization OR TI somatisation OR AB somatisation |
| S595 | TI Somatoform OR AB Somatoform |
| S596 | S593 OR S594 OR S595 |
| S597 | S 462 OR S484 OR S493 OR S503 OR S515 OR S526 OR S560 OR S577 OR S592 OR S596 |
| S598 | DE “Health Behavior” |
| S599 | DE “Health Promotion" |
| S600 | DE "Contraceptive Devices" |
| S601 | DE "Risk Factors" |
| S602 | DE "Protective Factors" |
| S603 | DE "Self-Examination (Medical)" |
| S604 | DE "Treatment Compliance" |
| S605 | DE "Meditation" |
| S606 | DE "Safe Sex" |
| S607 | DE "Social Behavior" |
| S608 | DE "Health Education" |
| S609 | DE "Lifestyle" |
| S610 | S598 OR S599 OR S600 OR S601 OR S602 OR S603 OR S604 OR S605 OR S606 OR S607 OR S608 OR S609 |
| S611 | TI nutrition OR AB Nutrition |
| S612 | TI "body weight" OR AB "body weight" |
| S613 | TI "weight reduction" OR AB "weight reduction" |
| S614 | TI Diet OR AB Diet |
| S615 | TI "weight loss" OR AB "weight loss" |
| S616 | TI "Food Intake" OR AB "Food Intake" |
| S617 | TI "Food consumption" OR AB "Food consumption" |
| S618 | TI "Nutritional Status" OR AB "Nutritional Status" |
| S619 | TI "Food preferences" OR AB "Food preferences" |
| S620 | TI "Food Habits" OR AB "Food habits" |
| S621 | S611 OR S612 OR S613 OR S614 OR S615 OR S616 OR S617 OR S618 OR S619 OR S620 |
| S622 | TI Exercise OR AB Exercise |
| S623 | TI sport OR AB sport OR TI sport OR AB sport |
| S624 | TI "Physical Fitness" OR AB "Physical Fitness" |
| S625 | TI "Physical Education" OR AB "Physical Education" |
| S626 | TI "Physical exertion" OR AB "Physical exertion" |
| S627 | TI "Movement therapy" OR AB "Movement therapy" |
| S628 | S622 OR S623 OR S624 OR S625 OR S626 OR S627 |
| S629 | TI "Health Education" OR AB "Health Education" |
| S630 | TI "Disease Control" OR AB "Disease Control" |
| S631 | TI Preven* OR AB Preven* |
| S632 | TI "Healthy People Program" OR AB "Healthy People Program" |
| S633 | TI "Patient Satisfaction" OR AB "Patient Satisfaction" |
| S634 | TI "wellness program" OR AB "wellness program" |
| S635 | TI "Obesity Management" OR AB "Obesity Management" |
| S636 | TI "sleep hygiene" OR AB "sleep hygiene" |
| S637 | TI "Self Care" OR AB "Self Care" |
| S638 | TI "Self-Care" OR AB "Self-Care" |
| S639 | TI "Patient adherence" OR AB "Patient adherence" |
| S640 | TI "Patient Compliance" OR AB "Patient Compliance" |
| S641 | S629 OR S630 OR S631 OR S632 OR S633 OR S634 OR S635 OR S636 OR S637 OR S638 OR S639 OR S640 |
| S642 | S610 OR S621 OR S628 OR S641 |
| S643 | S444 OR S597 OR S642 |
| S644 | S95 AND S404 AND S643 |
